# Polyunsaturated fatty acids reduce Fatty Acid Synthase and Hydroxy-Methyl-Glutaryl CoA-Reductase gene expression and promote apoptosis in HepG2 cell line

**DOI:** 10.1186/1476-511X-10-10

**Published:** 2011-01-18

**Authors:** Maria Notarnicola, Caterina Messa, Maria G Refolo, Valeria Tutino, Angelica Miccolis, Maria G Caruso

**Affiliations:** 1 Laboratory of Biochemistry, National Institute for Digestive Diseases, Castellana Grotte (BA), Italy

## Abstract

**Background:**

*n*-3 and *n*-6 polyunsaturated fatty acids (PUFAs) are the two major classes of PUFAs encountered in the diet, and both classes of fatty acids are required for normal human health. Moreover, PUFAs have effects on diverse pathological processes impacting chronic disease, such as cardiovascular and immune disease, neurological disease, and cancer.

**Aim:**

To investigate the effects of eicosapentaenoic acid (EPA) and arachidonic acid (ARA) on the proliferation and apoptosis of human hepatoma cell line HepG2 after exposure to increasing concentrations of EPA or ARA for 48 h. Moreover, in the same cells the gene expression of Fatty Acid Synthase (FAS) and 3-Hydroxy-3-Methyl-Glutaryl Coenzyme A Reductase (HMG-CoAR) was also investigated.

**Method:**

Cell growth and apoptosis were assayed by MTT and ELISA test, respectively after cell exposure to increasing concentrations of EPA and ARA. Reverse-transcription and real-time PCR was used to detect FAS and HMG-CoAR mRNA levels in treated cells.

**Results:**

Our findings show that EPA inhibits HepG2 cell growth in a dose-dependent manner, starting from 25 μM (P < 0.01, one-way ANOVA test and Dunnett's post test) and exerts a statistically significant pro-apoptotic effect already at 1 μM of EPA. Higher doses of ARA were need to obtain a statistically significant inhibition of cell proliferation and a pro-apoptotic effect in these cells (100 μM, P < 0.01, one-way ANOVA test and Dunnett's post test). Moreover, a down-regulation of FAS and HMG-CoAR gene expression was observed after EPA and ARA treatment in HepG2 cells, starting at 10 μM (P < 0.05, one-way ANOVA test and Dunnett's post test).

**Conclusion:**

Our results demonstrate that EPA and ARA inhibit HepG2 cell proliferation and induce apoptosis. The down-regulation of FAS and HMG-CoAR gene expression by EPA and ARA might be one of the mechanisms for the anti-proliferative properties of PUFAs in an *in vitro *model of hepatocellular carcinoma.

## Introduction

Hepatocellular carcinoma (HCC) is the fifth most prevalent malignancy worldwide, furthermore its incidence is rising [1,2]. In spite of recent progress in early diagnosis and curative transplantation or resection due to surveillance programs, most individuals present with advanced disease [3]. Limited non curative treatment options exist for such patients. Ethanol ablation, radiofrequency ablation, transarterial chemoembolization and selective radiation of lesions are some effective treatment options [3]. Systemic chemotherapy and other treatments such as external radiation, interferon, tamoxifen, antiandrogenic therapy or octreotide are ineffective and do not impact survival [4].

Recently, Gao et al. [5] have demonstrated the *in vitro *efficacy of C75, a Fatty Acid Synthase (FAS) inhibitor that induces growth arrest in several HCC derived cell lines, offering a promising novel therapy that explores another frontier for hepatocellular cancer treatment, i.e. metabolic manipulation. In addition to chemically synthesized compounds, much effort has been directed to identifying endogenous mechanisms of tumor suppression and, by high-throughput screening, of anticancer natural products. We have been studied some of these compounds, such as genistein, n6-isopenteniladenosine, polyphenols of olive oil, showing to possess anticancer properties *in vitro *and to modulate the action of lipogenic enzymes, such as FAS, 3-Hydroxy-3-Methyl-Glutaryl Coenzyme A Reductase (HMG-CoAR) or Farnesyl Pyrophosphate Synthase [6-8]. FAS is the key enzyme for the novo fatty acid synthesis [9]. Following a rate limiting step catalyzed by acetyl CoA carboxylase, it leads to the synthesis of palmityl CoA (a C16 long chain fatty acid). FAS is minimally expressed in most normal human tissues except the liver and adipose tissue, where it is expressed at high levels [10]. FAS expression is markedly increased in several human cancers compared with the corresponding normal tissue and its overexpression in tumors has been associated with a poor prognosis [11,12]. Our group has demonstrated an up-regulation of FAS in human colorectal cancer [13]. Enhanced FAS expression is a poor prognostic factor in patients with breast, prostate, and ovarian cancer, generating an intriguing paradigm that cancers with high expression of FAS have a growth and/or survival advantage and selective manipulation of FAS may be novel therapeutic strategy [14].

Cancer cells seem to require an increased concentration of cholesterol and cholesterol precursors and this requirement may be fulfilled by increased HMG-CoAR activity. In previous study, HMG-CoAR activity was found to be enhanced in human colorectal cancer that did not express LDL receptors, indicating that LDL receptors absence, which deprives colonic neoplastic cells of exogenous sterols, is overcome by an increase in endogenous cholesterol synthesis [15,16].

It is largely known that Polyunsaturated Fatty Acids (PUFAs) are potent inhibitors of both enzymes, HMG-CoAR and FAS [17-19] and they have been shown to have tumoricidal action, *in vitro *and *in vivo *[20]. Moreover, it is widely recognized that both *n*-3 and *n*-6 play additional roles as signaling molecules and modulators of lipogenic enzyme gene expression in liver [21], although epidemiological and experimental reports attribute antitumor properties above all to *n*-3 PUFAs [22-24]. PUFAs are natural constituents of animal and vegetable lipids. Long-chain PUFAs may be directly consumed in the diet or synthesized from their essential fatty acid precursors, linoleic acid and alfa-linolenic acid [25]. PUFAs have effects on diverse phathological processes impacting chronic disease, such as cardiovascular and immune disease, neurological disease and cancer. Clinical studies from cardiovascular medicine, and other disciplines have demonstrated correlations between low *n*-3 PUFA levels and increased disease risk and have shown that increasing *n*-3 levels by diet or supplementation may confer a variety of health benefits [26,27].

Our recent study [19] showed that eicosapentaenoic acid (EPA) significantly inhibited HMGCoAR gene expression and up-regulated mRNA LDL receptor in HepG2 cells and the combined treatment with EPA and Lovastatin enhanced the regulatory effect on gene expression of HMGCoAR and LDL receptor in the same cells. Moreover, we detected a synergistic effect on the inhibition of cancer cell proliferation obtained by combination of EPA and Lovastatin.

On the basis of these experimental data and in an effort to elucidate another aspect of PUFAs cell growth control, here we investigated the effects of EPA and arachidonic acid (ARA) on the proliferation and apoptosis of human hepatoma cell line HepG2. Moreover, in the same treated cells the gene expression of FAS and HMG-CoAR was also investigated.

## Materials and methods

### Cell culture conditions

HepG2, a cell line derived from human liver tissue with a well differentiated hepatocellular carcinoma, were obtained from the ICLC ( IST, Genoa, Italy).

Cells were routinely cultured in DMEM ( Dulbecco's modified Eagle's medium) supplemented with 10% FBS (fetal bovine serum ), 100 U/ml penicillin, 100 g/ml streptomycin, in monolayer culture, and incubated at 37°C in a humidified atmosphere containing 5% CO_2 _in air. At confluence, the grown cells were harvested by means of trypsinization and serially subcultured with a 1:4 split ratio. All cell culture components were purchased from Sigma-Aldrich (Milan, Italy).

### EPA and ARA treatment

To elucidate the effect of EPA and ARA on HMG-CoAR and FAS gene expression and cell growth, HepG2 cells were plated at a density of 3 × 10^5 ^cells/5 ml of DMEM containing 10% FBS in 60-mm tissue culture dishes (Corning Costar Co., USA).

Separate plates were seeded for each assay and when the cells were approximately 60% confluent were exposed to the treatment.

To examine the response to EPA and ARA, HepG2 cells were treated for 48 h with culture medium supplemented with various concentrations of EPA and ARA, separately (1, 10, 25, 50, 80 and 100 μM). Each experiment included a control without EPA or ARA and a control with the same amount of DMEM-BSA used for dissolving the fatty acid.

Triplicate culture were set up for each compound concentration and for control, and each experiment was repeated 4 times. Cell viability, determined using the trypan blue exclusion test, always exceeded 90%.

### Assessment of cell proliferation

After EPA and ARA treatment for 48 hours, the proliferative response on HepG2 was estimated by colorimetric 3-(4,5 di-methylthiazol-2-yl)-2,5-diphenyltetrazolium bromide (MTT) test. In brief, MTT stock (5 mg/ml in medium) was added to each dish at a volume of one-tenth the original culture volume and incubated for 2 hours at 37°C in humidified CO_2_. At the end of the incubation period, the medium was removed, and blue formazan crystal were solubilized with acidic isopropanol (0.1 N HCl in absolute isopropanol). MTT conversion to formazan by metabolically viable cells was monitored by spectrophotometer at an optical density of 570 nm.

### Assessment of cell apoptosis

The cytosolic DNA-histone complexes generated during apoptotic DNA fragmentation in treated HepG2 cells were evaluated with a cell death detection enzyme-linked immunosorbent assay (ELISA) kit (Roche Diagnostic GmbH, Mannheim, Germany) following the supplier's instructions.

### HMG-CoAR and FAS gene expression analysis

Analysis of gene expression was performed in HepG2 cells treated with 1, 10, 25, 50, 80 and 100 μM of EPA and ARA, separately for 48 hours.

Cells were washed twice in phosphate buffered saline (PBS) and then trypsinized and centrifuged at low speed. The cell pellets were resuspended in 0.3 ml pure distilled water and used for RNA extraction.

Total cell RNA was isolated with TRI-Reagent (Mol. Res. Centre Inc. Cincinnati, USA), following the manufacturer's instruction. About 2 μg total cell RNA, extracted from both the control and treated cells, was used for cDNA synthesis. Reverse transcription (RT) was carried out in 20 μl of the final volume at 41°C for 60 min, using 30 pmol antisense primer (Table 1) for analyses of the HMGCoAR, FAS and β-actin gene. The β-actin gene was utilized as an internal control and was chosen as a reference gene because it is a housekeeping gene.

**Table 1:** Sequences of amplification primers

Gene		Primer
HMG-CoAR	Sense	5'-TACCATGTCAGGGGTACGTC-3'
	Antisense	5'-CAAGCCTAGAGACATAATCATC
FAS	Sense	5'-TATGCTTCTTCGTGCAGCAGTT-3'
	Antisense	5'-GCTGCCACACGCTCCTCTAG-3'
β-actin	Sense	5'-AAAGACCTGTACGCCAACACAGTGCTGTCTGG-3'
	Antisense	5'-CGTCATACTCCTGCTTGCT GATCCACATCTGC-3'

Real-time PCRs were performed in 25 μl final volume containing 2 μl cDNA, master mix with SYBR Green (iQ SYBR Green Supermix; Bio-Rad, Milan, Italy) and sense and antisense primers for HMGCoAR, FAS and β-actin gene (Table 1).

Real-Time PCR was carried out with iCycler Thermal Cycler System apparatus (Bio-Rad) using the following parameters: one cycle of 95°C for 1 min and 30 s, followed by 45 cycles at 94°C for 10 s, 55°C for 10 s and 72°C for 30 s and a further melting curve step at 55-95°C with a heating rate of 0.5°C per cycle for 80 cycles. The PCR products were quantified by external calibration curves, one for each tested gene, obtained with serial dilution of known copy number of molecules (10^2^-10^7 ^molecules). All expression data were normalized by dividing the amount of target by the amount of β-actin used as internal control for each sample. The specificity of the PCR product of each tested gene was confirmed by gel electrophoresis.

### Statistical Analysis

The significance of the differences between the control group versus each experimental group of concentration was evaluated with one way analysis of variance (ANOVA) and the Dunnett' post test. Differences were considered significant at a 5% probability level.

## Results

**Figure 1:**
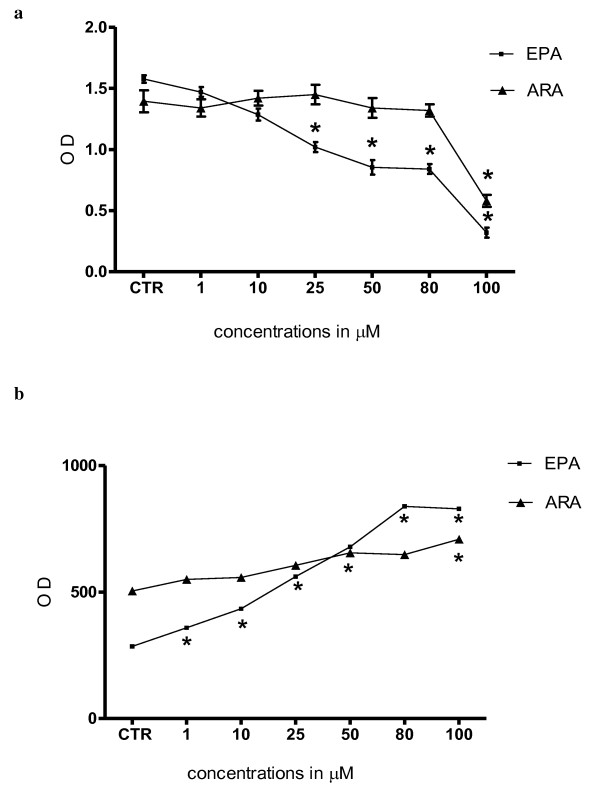
***Panel a*: Effect of increasing EPA and ARA concentrations on the conversion of MTT-tetrazolium salt in HepG2 cells and *Panel b: *Apoptosis in HepG2 cells after EPA and ARA treatment**. All data are the mean ± SE of four consecutive experiments. *P *value was determined by ANOVA with Dunnett' post test. *P < 0.01 versus control.

Our findings show that EPA inhibits HepG2 cell growth in a dose-dependent manner, starting from 25 μM (P < 0.01, one-way ANOVA test and Dunnett's post test) (Figure 1a) and exerts a statistically significant pro-apoptotic effect already at 1 μM (Figure 1b). Higher doses of ARA were need to obtain a statistically significant inhibition of cell proliferation and a pro-apoptotic effect in same cells (100 μM, P < 0.01, one-way ANOVA test and Dunnett's post test) (Figure 1a and 1b), likely being HepG2 cells more sensitive to EPA.

**Figure 2:**
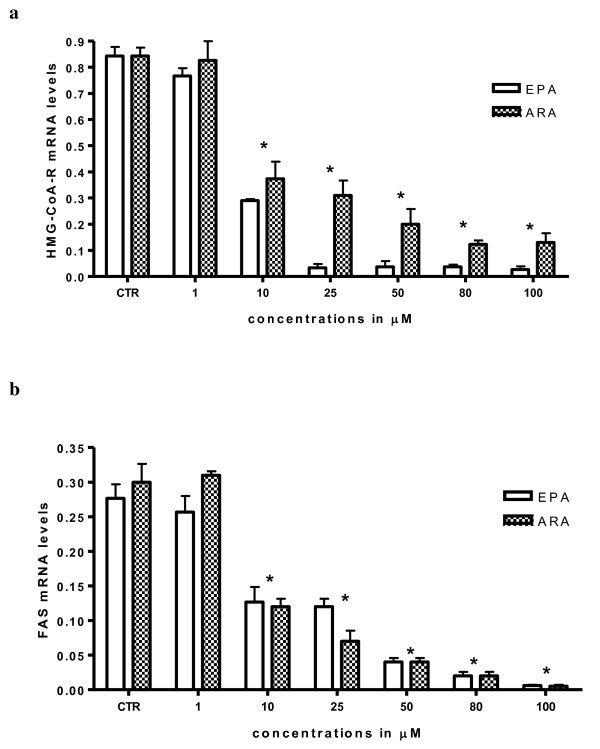
**EPA and ARA effects on HMG-CoAR (panel a) and FAS (panel b) mRNA levels in HepG2 cells**. All data represent the mean ± SE of four consecutive experiments. mRNA levels are expressed as ratio of the amount of gene target by the amount of β-actin. *P *value was determined by ANOVA with Dunnett' post test. *P < 0.05 versus control.

Moreover, a down-regulation of HMG-CoAR and FAS gene expression was observed after EPA and ARA treatment in HepG2 cells, starting at 10 μM (P < 0.05, one-way ANOVA test and Dunnett's post test) (Figure 2a and 2b).

The exogenous administration of 1 mM mevalonate to cells treated with PUFAs reverted the effect on HMGCoA reductase gene expression but not on FAS gene expression (data not shown).

## Discussion

Recently, research on natural diet-based agents for anti-cancer has been focused on activity capable of selective or preferential elimination of cancer cells by inhibiting cell growth and/or causing apoptosis [6-8].

In this study, we show that the exposure of HepG2 hepatoma cells to EPA and ARA leads to a cell growth arrest and promote the apoptosis. The inhibition of cell growth exerted by these PUFAs appears to be due to a strong inhibition of gene expression of FAS and HMG-CoAR. EPA inhibits FAS and HMG-CoAR gene expression in HepG2 cells more efficaciously than ARA, because lower doses of EPA are able to elicit the inhibitor effect. This finding suggests that both PUFAs affect growth and viability even if with different kinetics. The FAS and HMG-CoAR inhibition causes in the cell a reduction of lipids, such as fatty acids, cholesterol, both essential for the activity of cancer cells and its proliferation [13,15,16,28].

Our findings confirm the PUFAs anti-proliferative effects and demonstrate that the antiproliferative efficacy of EPA and ARA is due to down-regulation of lipogenic enzyme gene expression, such as HMG-CoAR and FAS. The efficacy in down-regulation of gene expression was detected at the same doses for both substances, but the cell growth inhibition and apoptosis was elicited at the lower doses for EPA than ARA, confirming a major efficacy for *n*-3 PUFA as anti-cancer agent.

Previous studies showed that PUFAs selectively induced tumor cells apoptosis though the sensitivity of various cancer cells to different fatty acids were found to be variable depending on the type of cancer cell being tested and the type and concentration of the fatty acid used [29,30]. Previously, it was reported that essential fatty acids and their metabolites suppress tumor cells growth both in vitro and in vivo, though at different concentrations [29]. It was also opined that *n-6 *fatty acids enhance tumor cell growth whereas *n-*3 fatty acids are beneficial since they arrest cell growth. This differential action of *n-3 *and *n-6 *PUFAs in cancer has been attributed to the formation of pro-inflammatory eicosanoids from *n-6 *PUFAs whereas products formed *n-3 *PUFAs are much less pro-inflammatory in nature [30].

Moreover, the present work shows that cell growth inhibition of HepG2 by EPA and ARA takes place by down-regulation of HMG-CoAR and FAS gene expression. Previous studies have revealed that PUFAs can regulate the expression of genes involved in several metabolic pathways [17-19]. It has been established that the lipogenic enzymes are in general regulated co-ordinately at mRNA level [31,32]. Our current finding that lipogenic enzymes such as FAS and HMG-CoAR are at the same time and with the same dose of PUFAs down-regulated in HepG2 cells is in agreement with this hypothesis. Although the precise molecular mechanism for this regulation is currently unclear, PUFAs may control lipogenic gene expression through their effects on SREBP-dependent regulation [33]. SREBP-responsive genes include those coding for HMG-CoAR and for intermediates in cholesterol synthesis, as Farnesyl Pirophosphate Synthase, and for FAS [21]. PUFAs decreased mRNA levels of lipogenic enzymes in rat hepatoma cells and mouse liver, in correlation with their effects on HMG-CoAR and FAS [24,34]. Fish oil feeding, rich in the *n-3 *PUFAs, drastically decreased mRNA levels for lipogenic enzymes in rodent liver [35].

It is also well known that several effects of PUFA are due to their ability in activating peroxisome proliferator-activated receptors (PPARs) as shown in human lung-tumor cells [36].

Since elevated expression of HMG-CoAR and FAS in malignant cells has been documented in various cancers including breast, prostate, colon stomach, ovarian, endometrial, lung and hepatocellular carcinoma, compounds that can inhibit these enzymes in tumor cells, therefore, may be effective as adjuvant chemoterapeutic agents. Inhibition of cell growth and HMG-CoAR and FAS gene expression was seen at concentrations of *n-3 and n-6 *PUFAs that were an order of magnitude very low. Dietary sources, therefore, may provide EPA and ARA at levels sufficient to regulate lipogenic enzymes, but an increased exogenous PUFAs administration should be necessary to modulate carcinogenesis process. In fact, caution must be applied when extrapolating in vitro results into clinical practice.

In conclusion, at least in the experimental model used throughout the present investigation, cell growth inhibition and apoptosis of hepatoma cells might be dependent of the PUFA-induced down-regulation of lipogenic enzyme gene expression, underlying a role for dietary components in neoplastic cell growth control.

## Competing interests

The authors declare that they have no competing interests.

## Authors' contributions

MN and MGC conceived the study, participated in its design and coordination. CM, MGR, VT, and AM, performed various experiments. MN and MGC interpreted the data and wrote the manuscript. all authors read and approved the final manuscript.
